# Experimental Study of Amphibolite–Basalt (SiO_2_-AlO_3_-CaO-Fe_2_O_3_) Glasses for Glass-Ceramic Materials Production

**DOI:** 10.3390/ma16216887

**Published:** 2023-10-27

**Authors:** Malgorzata Lubas, Anna Zawada, Jaroslaw Jan Jasinski, Adrian Nowak

**Affiliations:** 1 The Czestochowa University of Technology, Department of Materials Engineering, Armii Krajowej 19, 42-200 Czestochowa, Poland; anna.zawada@pcz.pl; 2 National Centre for Nuclear Research, Centre of Excellence NOMATEN, A. Soltana 7 St., 05-400 Otwock, Poland; jaroslaw.jasinski@ncbj.gov.pl; 3 The Czestochowa University of Technology, Doctoral School of CUT, Dabrowskiego 69, 42-200 Czestochowa, Poland; adrian.nowak@pcz.pl

**Keywords:** multicomponent glasses, pyroxenes, glass-ceramic materials, FTIR spectroscopy, mineral fibres

## Abstract

The paper presents research on multicomponent glasses obtained from natural and secondary raw materials, i.e., basalt, amphibolite, and cullet. The raw materials were used as potential sets to produce mineral fibres or glass-ceramic materials. FTIR spectroscopy and XRD studies were carried out to identify the composition of the phase type in the glass sets. The results were supported by SEM-EDS microstructural studies of the obtained materials. The ability of the melts to crystallize and their basic properties required in producing mineral fibres, i.e., the hardness and the acidity modulus, were also determined. In the glass samples after the crystallization process, the spectroscopic studies revealed an increase in the half-width of the band at 1200–800 cm^−1^ and splitting at the values of about 870 cm^−1^ and 970 cm^−1^. These changes probably indicate the formation of pyroxene-type crystalline phases. Moreover, based on the XRD results, it was confirmed that the obtained materials were fully amorphous. After annealing at 800 °C for 2 h, the materials show a small proportion of crystalline phases. For the materials annealed at higher temperatures, clear peaks from the crystalline phases were represented mainly by pyroxenes. The proportion of crystalline phases in the samples was also found to rise with increasing temperature, and the hardness values for the basalt glasses and glasses after crystallization rose from 753 to 946 HV0.05. Such an effect positively affects the properties of the obtained glass-ceramic materials based on the proposed sets. However, in the case of mineral fibres, crystallization at early 2 h at 800 °C can be a disadvantageous feature from the point of view of their application because crystalline phases can lead to fibre damage after a short period of operation; this will be confirmed in this study.

## 1. Introduction

The fast development of glass materials technology and more innovative solutions in building insulation systems to obtain adequate acoustic levels, construction fire, and thermal insulation requires using novel mineral fibre elements and manufacturing new glass or glass-ceramic materials [1,2,3]. The most commonly used material for mineral wool is basalt, which is a mafic, extrusive rock that makes up more than 90% of all volcanic rocks. It has a crystalline structure that changes depending on the specific conditions of the lava flow. Basalts mainly consist of three minerals—pyroxene, olivine, and plagioclase [4,5]. This raw material is mainly used as road aggregate and for producing plates and tiles, linings for steel pipes, etc. In recent years, basalts have also been used to produce bricks and mineral basalt fibres, which, recently, with great success, have even found architectural applications as well. Mineral fibres are non-flammable, do not react toxically with water and air, are highly resistant to strong alkalis compared to glass fibres, and have even better mechanical and physical properties than carbon or silicon carbide fibres [5,6,7,8]. Mineral basalt fibres are used to produce materials that require resistance to high temperatures, acids, and solvents but that also need to be durable, mechanically robust, and exhibit low absorbency [9,10,11]. Mineral fibres are mainly produced using cupola technology and blowing/duplex. Basalt fibre production technology is similar to glass fibres but requires less energy. The abundant availability of raw materials worldwide makes it possible to achieve lower production costs than glass fibres [10]. The modification of mineral fibres by using mixtures containing other mineral raw materials or secondary ones (for example, amphibolite, gabbro, glass cullet, fly ash, and blast furnace slag) allows the technological requirements of the defibering process to be met and ensures the quality of the produced fibres. However, for fibres to meet their functions, they must complete basic requirements, such as the appropriate chemical composition of the set and the resulting melt, which should be homogeneous, with optimal viscosity, which is in the range of 0.5–2.5 Pa∙s during the defibering process, and a melting point that should be below 1500 °C [12,13,14,15]. On the other hand, glass-ceramic (GC) materials are inorganic silicate materials characterized by the presence of crystals corresponding to one or more phases embedded in a glass (amorphous) matrix. They are mainly obtained through the controlled crystallization of adequately prepared glass compositions [16]. Glass-ceramic products can also be produced through the controlled cooling of molten glass or through the sintering and crystallizing of glass powders. The latter method involves compacting the powders at relatively low temperatures in combination with viscous flow [17]. The literature shows that current technologies can convert complex chemical compositions (derived from minerals or silicate waste) into functional glass or glass-ceramic materials [18,19]. However, designing multicomponent sets to develop glass-ceramics requires careful chemical and phase composition control. They will determine the physicochemical and mechanical properties of the materials. In particular, oxides are required that act as network-forming substances, silicon oxide (SiO_2_) and phosphorus oxide (P_2_O_5_), as well as network-modifying oxides, such as sodium oxide (Na_2_O), potassium oxide (K_2_O), magnesium oxide (MgO), and calcium oxide (CaO). Also, oxides acting as crystallization nuclei are needed, e.g., titanium oxide (TiO_2_) or iron oxide (Fe_2_O_3_) [20]. According to Erol et al., a significant amount of Fe_2_O_3_ can be used as a nucleating agent in producing glass-ceramic materials, providing an essential advantage in developing a wide variety of microstructures with different morphological conformations [21,22]. 

This paper presents research on multicomponent melted basalt–amphibolite materials with the addition of packaging. It has been demonstrated that glass-ceramic materials formed from multicomponent sets containing waste raw materials have the potential to compete with products made exclusively from natural raw materials, especially considering mechanical properties [23]. Ayala Valderrama et al. [24] presented the results of a study in which they demonstrated the influence of crystalline phases on the mechanical properties of the obtained glass-crystalline materials. They showed that the presence of crystalline phases, such as diopside 73% and 27% anorthite, gives strengths of about 10 MPa. In contrast, other phases, such as enstatite, wollastonite, and anorthite, show lower strength values of glass-crystalline ceramics [25,26]. The authors of the work suggest that diopsite is the most favourable or preferred crystalline phase in CAS glass ceramics. ASTM C28 (2015) [27] indicates that values in the 3.1–8.3 MPa range are relevant for gypsum material applications in construction and concrete [28].

Recently, interesting research results have been presented regarding using granite waste to produce glass-ceramic materials, too. The authors [29] showed that the increase in the Si:O ratio causes the crystallization of wollastonite, not diopside. For 40% granite waste content, the crystal structure of glass ceramics is moderate, the bulk density is 3.0207 g/cm^3^, and the microhardness is 8.6 GPa, which shows that the obtained glass-ceramic materials are perfectly suitable for architectural decorations. Luo et al. [30] used granite waste as the primary raw material for producing glass-ceramics with a bending strength above 200 MPa. However, the production of glass-ceramics required the use of nucleating agents. Using these agents undoubtedly increases the cost of glass-ceramics production and reduces its commercial profitability, transportation, and stone processing. In the case of a glass cullet, it can be recycled entirely to exploit alternatives to its final disposal. They could be used as additives in asphalt and concrete production [31,32]. As a technical alternative, the author’s research works have glass cullet used in obtaining glass-ceramics, bearing in mind that these materials may have attractive properties for applications in several industrial sectors.

Additionally, glass-ceramic technology represents a versatile (materials science) approach to immobilizing various radioactive and dangerous wastes. However, due to the high melting temperatures and special heat treatment conditions required to reach adequate properties of glass-ceramics, waste can only be justified if high-value products with suitable properties for industrial applications can be developed [33,34]. Savvilotidou et al. [35] presented a study that investigates an innovative approach for valorizing specific wastes generated from the energy sector and producing glass-ceramics. The wastes used were photovoltaic (P/V) glass, produced from the renewable energy sector, and lignite fly ash, produced from the conventional energy sector. The process first involved the production of glass after melting specific mixtures of wastes, namely (i) 70% P/V glass and 30% lignite fly ash and (ii) 80% P/V glass and 20% lignite fly ash at 1200 °C for 1 h, as revealed through the use of a heating microscope. The results indicated that the P/V glass, as a sodium–potassium-rich inorganic waste, reduces the energy requirements of the melting process. The produced glass was then used for the production of glass-ceramics. Dense and homogeneous glass-ceramics, exhibiting high chemical stability and no toxicity, were produced after controlled thermal treatment of glass at 800 °C. The properties of the produced glass-ceramics (namely, water absorption and compressive strength) render them suitable for applications in the construction industry. The waste valorization approach followed in this study aligns with the circular economy principles. The possibility of using the proposed glass sets to produce mineral fibres or glass-ceramic materials was verified through a glass melting process in an electric furnace at 1450 °C for 2 h and determination of fundamental properties, such as the crystallization ability, microstructure, mechanical properties, and the acidity modulus. 

## 2. Materials and Methods

The materials used in this study were melted basalt–amphibolite samples modified with a colorless glass cullet. Four sets of raw materials were prepared with the contents shown in Table 1.

The prepared raw material sets were firstly homogenized and melted in an electric furnace at a temperature of 1450 °C for 2 h to obtain homogeneous glass melts. The obtained melts were cast directly onto a steel plate to ensure a high cooling rate. The chemical composition of the obtained glasses was determined through XRF spectroscopy. This study was performed using a WDXRF Axios, Malvern Panalytical Ltd., Malvern, United Kingdom; spectrometer with a 4 kW Rh lamp, and the results are shown in Table 2.

Dilatometric studies were performed for the melted samples using a Sadamel DA-3, automatic dilatometer (SADAMEL SA, La Chaux-de-Fonds, Switzerland), allowing T_g_ transformation temperature to be determined. Structural studies were carried out using FTIR spectroscopy and X-ray diffraction. IR spectra in the 400–4000 cm^−1^ range were obtained with a Fourier spectrometer (Bruker Optics-Vertex 70V, Billerica, MA, USA). Measurements were made employing the powder technique, and the absorption of the spectrum was recorded with 128 scans and a resolution of 4 cm^−1^. The X-ray studies were performed on a SEIFERT XRD-3003 T-T X-ray diffractometer (XRD Eigenmann GmbH, Schnaittach, Germany) with a lamp with a wavelength of λ_Co_ = 0.17902 nm at the angle range of 2ϴ 5–90°. Microstructural studies were conducted employing a Keyence VHX-7000N digital microscope (Keyence, Osaka, Japan) while scanning electron microscopy SEM was performed using a JOEL JSM-6610LV microscope (Peabody, MA, USA). As part of the research, microhardness tests were also performed (HV0.05). Hardness was measured using a Shimadzu HMV-G20 (Kioto, Japan) microhardness tester with a Vickers indenter. For each sample, 10 measurements in different areas of the sample were carried out, and then the average microhardness value was determined.

## 3. Results and Discussion

### Study of Amphibolite Glasses after the Melting Process

**Figure 1:**
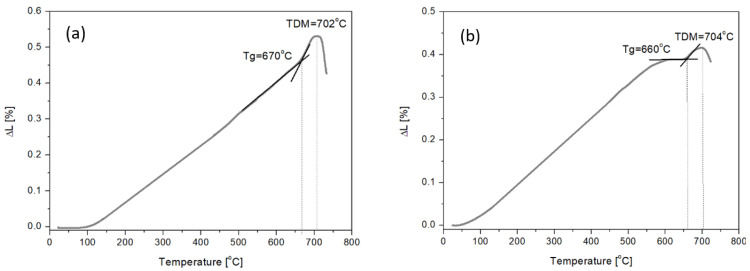

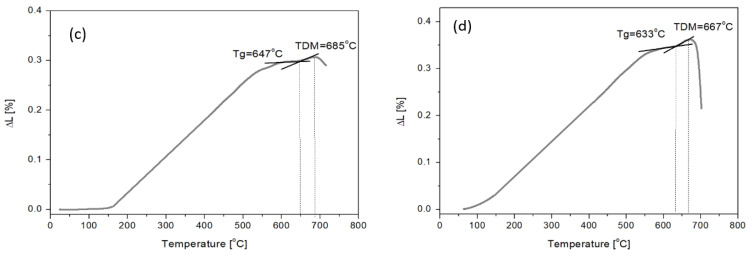
Dilatometric curves of SiO_2_-Al_2_O_3_-CaO-MgO-Fe_2_O_3_ multicomponent glasses: (**a**) glass 1—100 wt.% basalt, (**b**) glass 2—50 wt.% basalt, 40 wt.% amphibolite, 10 wt.% cullet, (**c**) glass 3—50 wt.% basalt, 30 wt.% amphibolite, 20 wt.% cullet, (**d**) glass 4—50 wt.% basalt, 20 wt.% amphibolite, 30 wt.% cullet.

The first stage of this study was dilatometric tests to determine transformation temperature T_g_ and softening temperature DTM for the obtained melts. The glass transition (Tg) in dilatometric tests corresponds to the inflection of the curve with a significant change in dimensions (according to ASTM E1545 [36]). The results in the form of curves are shown in Figure 1.

**Figure 2:**
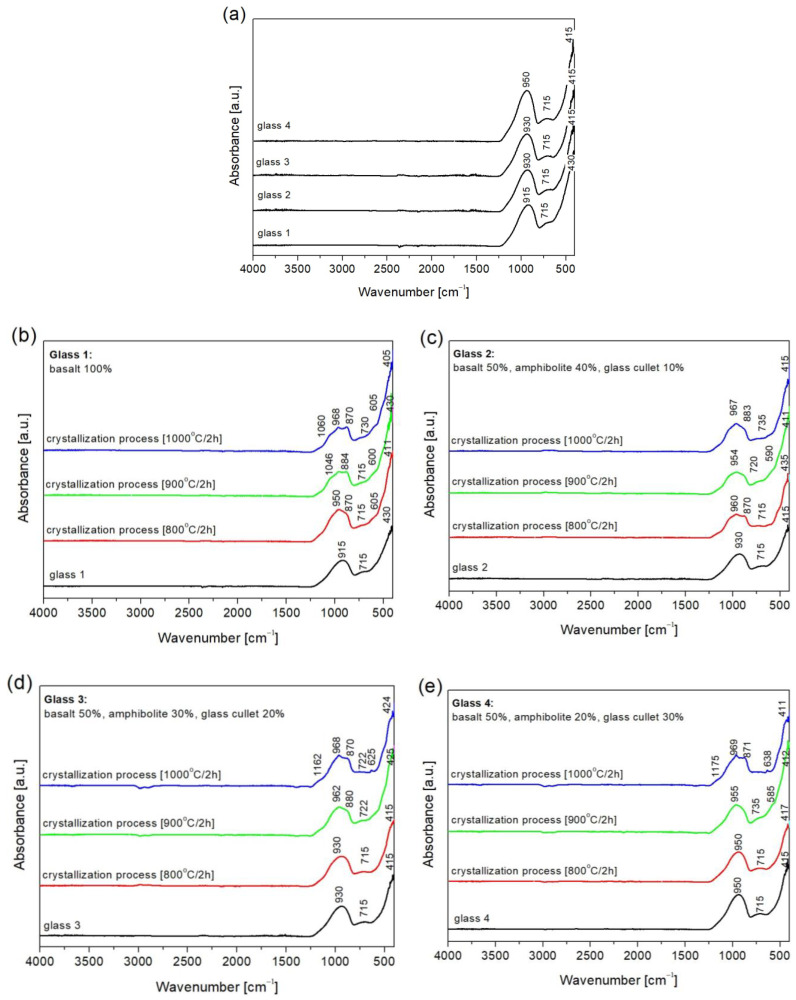
FTIR spectra of SiO_2_-Al_2_O_3_-CaO-MgO-Fe_2_O_3_ multicomponent glasses: (**a**) glass 1–4; (**b**) glass 1—100% basalt; (**c**) glass 2—50 wt.% basalt, 40 wt.% amphibolite, 10 wt.% cullet; (**d**) glass 3—50 wt.% basalt, 30 wt.% amphibolite, 20 wt.% cullet; (**e**) glass 4—50 wt.% basalt, 20 wt.% amphibolite, 30 wt.% glass cullet—after crystallization process.

Based on the results, it was found that the modification of basalt–amphibolite alloys by adding a cullet lowers the transformation temperature. When analyzing the chemical composition, it was found that as the cullet content increases and the proportion of amphibolite decreases, the proportion of Na_2_O oxide increases, which acts as a flux that affects the reduction of the transformation temperature from 670 °C (Set 1) to 633 °C (Set 4). The next stage of the research was performed for samples after melting, and the crystallization process was carried out at temperatures 800 °C, 900 °C, and 1000 °C for 2 h. Spectroscopic studies performed for structure determination of the obtained materials showed that melts contain alkali ions in their structure (Table 2), which contribute to the depolymerization of the lattice, i.e., the breaking of Si-O-Si bridge bonds, resulting in the growth of non-bridging Si-O- bonds [37]. The melted samples (Figure 2a) are characterized by three prominent absorption bands, where their maxima are located at about 940, 715, and 411 cm^−1^. The most intense absorption bands in the 1200–800 cm^−1^ range can come from the vibration of Si-O-(Al) and Si-O-(Si) bridges. The absorption bands in the 800–650 cm^−1^ range, characterized by lower intensity, come from symmetric bending vibrations of Si-O-Si. The 650–400 cm^−1^ band corresponds to O-Al-O and O-Si-O bending vibrations [38,39,40].

In turn, spectroscopic studies showed that the samples subjected to the crystallization process demonstrate changes in the spectra (Figure 2b–e). In addition to the distinguished three prominent absorption bands in the 1200–800 cm^−1^, 800–600 cm^−1^, and 600–400 cm^−1^ ranges, an increase in the half-width of the band in the 1200–800 cm^−1^ range was observed, which confirms the depolymerization of the glass bond—a reduction in the number of Si-O-Si, Si-O-Al bonds [40]. The band at about 870 cm^−1^ and 970 cm^−1^ is divided into two. The change in the width of the bands and their separation may indicate the presence of crystalline phases formed as a result of the conducted crystallization process, thereby affecting the change in structure. This may indicate the formation of crystalline phases in the crystallization process of glasses, mainly pyroxenes (diopside), whose bands are at a maximum of 865 cm^−1^ [39,41].

**Figure 3:**
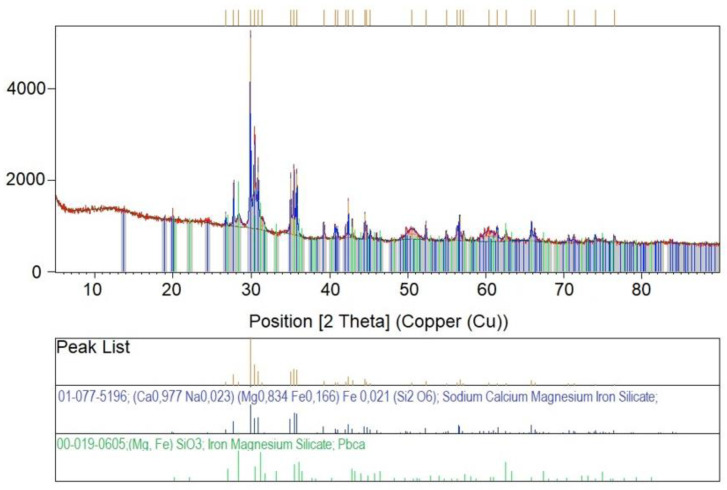
X-ray diffraction patterns, theoretical and experimental, for glass 1—100% basalt (1000 °C/2 h).

**Figure 4:**
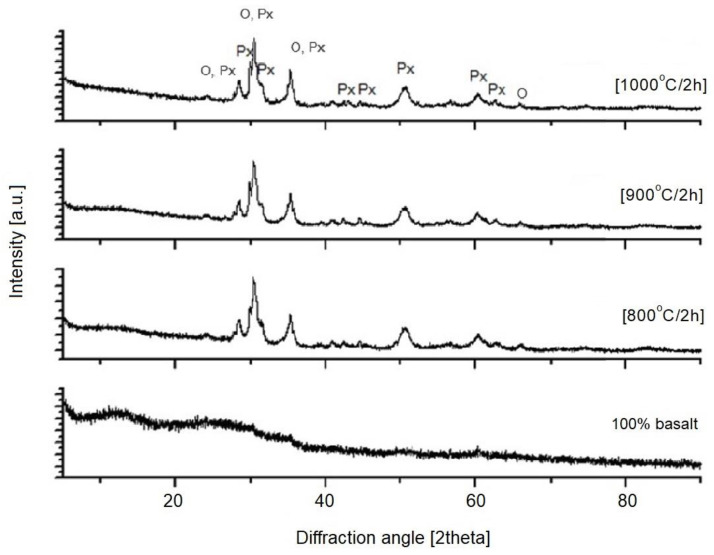
X-ray diffraction patterns of SiO_2_-Al_2_O_3_-CaO-MgO-Fe_2_O_3_ multicomponent glass (1—100 wt.% basalt), after melting and crystallization process; Px—pyroxenes, O—olivines.

**Figure 5:**
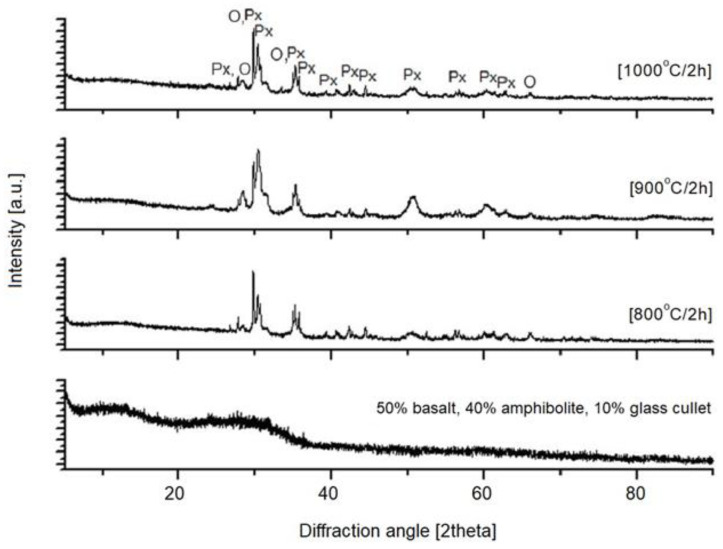
X-ray diffraction patterns of SiO_2_-Al_2_O_3_-CaO-MgO-Fe_2_O_3_ multicomponent glass (2—50 wt.% basalt, 40 wt.% amphibolite, 10 wt.% cullet), after melting and crystallization process; Px—pyroxenes, O—olivines.

**Figure 6:**
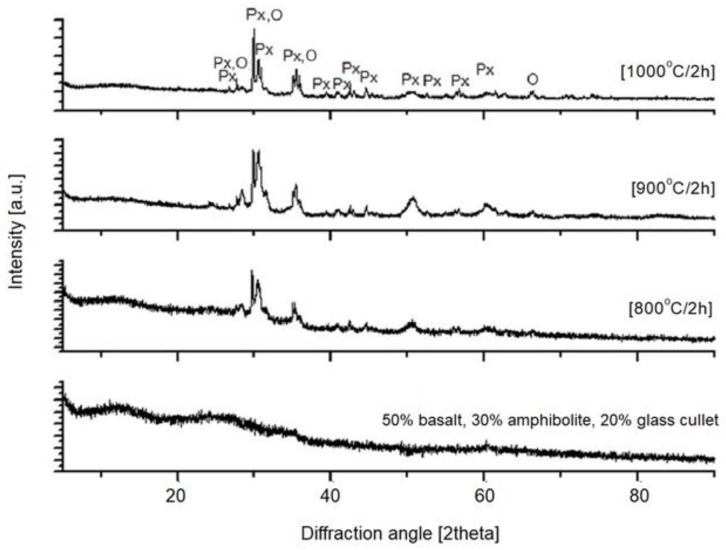
X-ray diffraction patterns of SiO_2_-Al_2_O_3_-CaO-MgO-Fe_2_O_3_ multicomponent glass (3—50 wt.% basalt, 30 wt.% amphibolite, 20 wt.% glass cullet), after melting and crystallization process; Px—pyroxenes, O—olivines.

**Figure 7:**
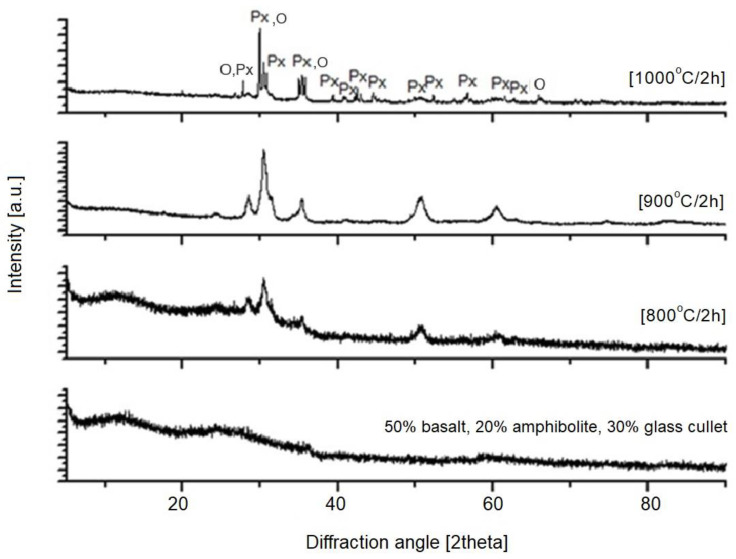
X-ray diffraction patterns of SiO_2_-Al_2_O_3_-CaO-MgO-Fe_2_O_3_ multicomponent glass (4—50 wt.% basalt, 20 wt.% amphibolite, 30 wt.% glass cullet), after melting and crystallization process, Px—pyroxenes, O—olivines.

The next step in this study was X-ray diffraction (XRD) phase analysis. The results obtained for the glasses after melting and the crystallization process are shown in Figure 3, Figure 4, Figure 5, Figure 6 and Figure 7. As the same crystalline phases were present in all of the analyzed glasses, one example XRD result is included in this paper, with reference data for basalt glass annealed at 1000°/2 h (Figure 3).

The XRD results found that the materials received after the melting process do not show any peaks, indicating the participation of crystalline phases. Only an elevation of the background in the range of 20–35° 2Θ is visible, which indicates the amorphous structure of the obtained materials. The glasses subjected to crystallization showed the presence of peaks originating from the forming crystalline phases. The proportion of crystalline phases at lower processing temperatures is insignificant. The diffractograms for the materials annealed at higher temperatures show clear, sharp peaks originating from crystalline phases, mainly represented by pyroxenes and olivines. The obtained materials are characterized by a high content of Ca^2+^, Mg^2+^ and Fe^+^, Al ions (Table 2), which explains the crystallization of pyroxene phases (diopside, augite type) [42], as confirmed by the results of spectroscopic studies too. The XRD results determined the proportion of amorphous and crystalline phases for the analyzed glasses undergoing crystallization. Rietveld analysis was used for this purpose. The results obtained are summarized in Table 3.

**Figure 8:**
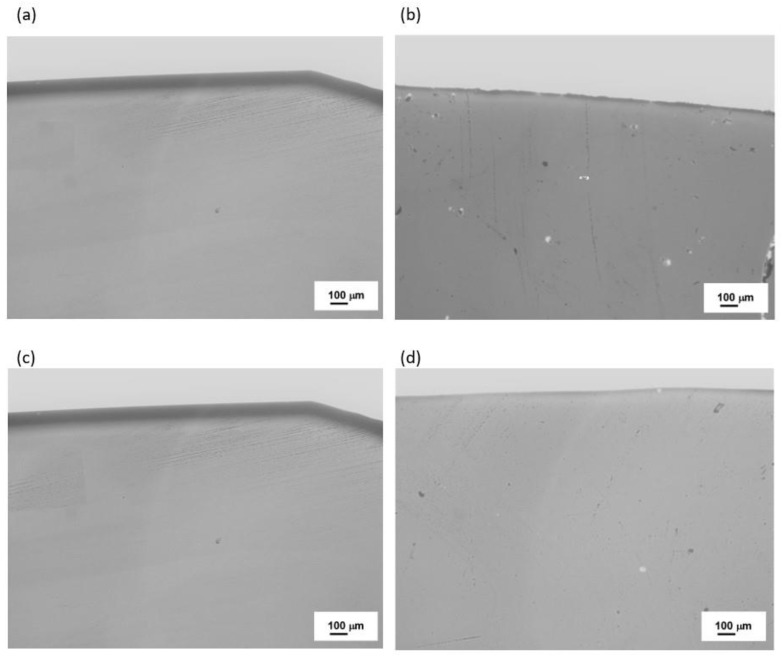
Microstructure (Optical Microscopy) of SiO_2_-Al_2_O_3_-CaO-MgO-Fe_2_O_3_ multicomponent glasses after melting: (**a**) glass 1—100 wt.% basalt; (**b**) glass 2—50 wt.% basalt, 40 wt.% amphibolite, 10 wt.% cullet; (**c**) glass 3—50 wt.% basalt, 30 wt.% amphibolite, 20 wt.% cullet; (**d**) glass 4—50 wt.% basalt, 20 wt.% amphibolite, 30 wt.% cullet.

**Figure 9:**
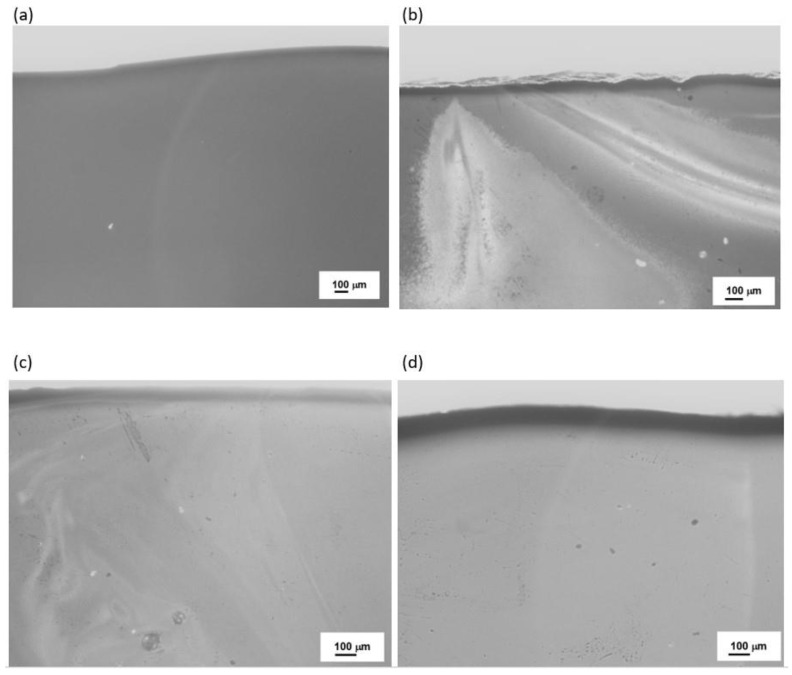
Microstructure (Optical Microscopy) of SiO_2_-Al_2_O_3_-CaO-MgO-Fe_2_O_3_ multicomponent glasses after crystallization process at 800 °C for 2 h: (**a**) glass 1—100 wt.% basalt; (**b**) glass 2—50 wt.% basalt, 40 wt.% amphibolite, 10 wt.% cullet; (**c**) glass 3—50 wt.% basalt, 30 wt.% amphibolite, 20 wt.% cullet; (**d**) glass 4—50 wt.% basalt, 20 wt.% amphibolite, 30 wt.% cullet,.

**Figure 10:**
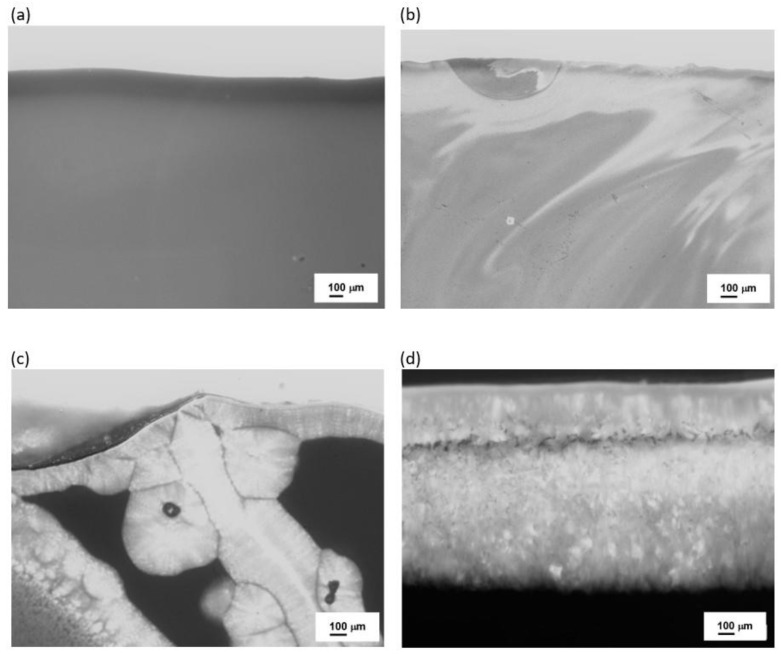
Microstructure (Optical Microscopy) of SiO_2_-Al_2_O_3_-CaO-MgO-Fe_2_O_3_ multicomponent glasses after crystallization process at 900 °C for 2 h: (**a**) glass 1—100% basalt; (**b**) glass 2—50 wt.% basalt, 40 wt.% amphibolite, 10 wt.% cullet; (**c**) glass 3—50 wt.% basalt, 30 wt.% amphibolite, 20 wt.% cullet; (**d**) glass 4—50 wt.% basalt, 20 wt.% amphibolite, 30 wt.% cullet.

**Figure 11:**
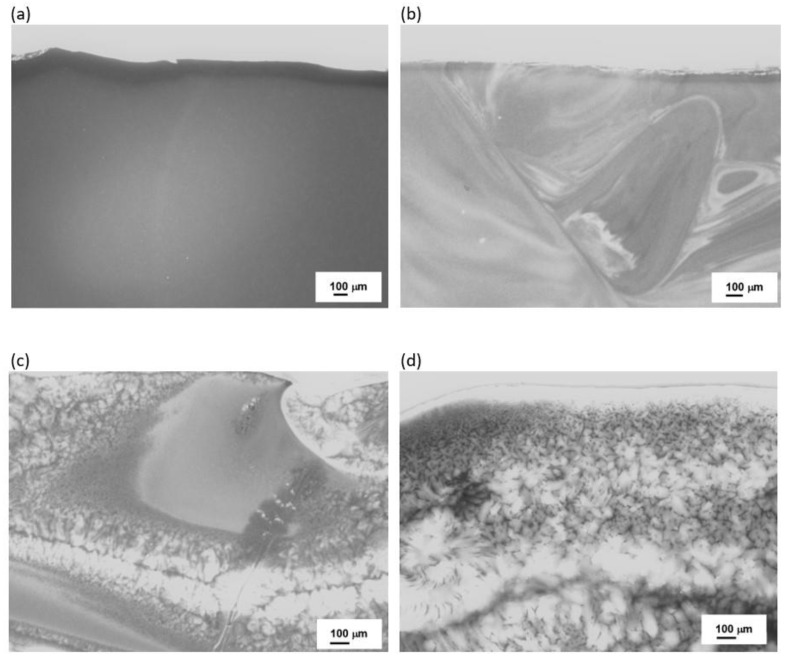
Microstructure (Optical Microscopy) of SiO_2_-Al_2_O_3_-CaO-MgO-Fe_2_O_3_ multicomponent glasses after crystallization process at 1000 °C for 2 h: (**a**) glass 1—100% basalt; (**b**) glass 2—50 wt.% basalt, 40 wt.% amphibolite, 10 wt.% cullet; (**c**) glass 3—50 wt.% basalt, 30 wt.% amphibolite, 20 wt.% cullet; (**d**) glass 4—50 wt.% basalt, 20 wt.% amphibolite, 30 wt.% cullet.

To confirm these findings, further microscopic observations (Digital Microscopy and SEM/EDS) were performed to determine changes in the microstructures of the analyzed samples before and after the crystallization process. The results are shown in Figure 8, Figure 9, Figure 10 and Figure 11.

**Figure 12:**
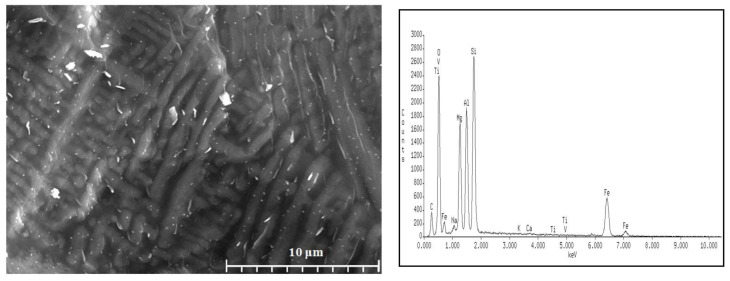
Microstructure (SEM) and EDS analysis of SiO_2_-Al_2_O_3_-CaO-MgO-Fe_2_O_3_ multicomponent glass after crystallization process by annealing at 1000 °C for 2 h: glass 4—50 wt.% basalt, 20 wt.% amphibolite, 30 wt.% glass cullet.

No crystalline phases were observed in the microstructure of the melted samples for any of the sets from 1 to 4 (Figure 8a, Figure 9a, Figure 10a, and Figure 11a). The samples produced from Set 2 (50 wt.% basalts, 40 wt.% amphibolite, 10 wt.% glass cullet) annealed at 800 °C for 2 h (Figure 8b, Figure 9b, Figure 10b, and Figure 11b) show the presence of crystalline phases, while Set 3 (50 wt.% basalt, 30 wt.% amphibolite, 20 wt.% glass cullet) and Set 4 (50 wt.% basalt, 20 wt.% wt. amphibolite, 30 wt.% glass cullet) show opacity and demixing as a first stage crystallization. Annealing the samples at 900 °C (Figure 10a–d) and 1000 °C (Figure 11a–d) for 2 h results in the apparent crystalline phases. Upon increasing the annealing temperature and time, a rise in the proportion of crystalline phases and volumetric crystallization of the glasses was observed. Microstructure studies were also performed using SEM-EDS to identify the crystalline phases fully. Figure 12 shows an SEM-EDS image for glass Set 4 (50 wt.% basalt, 20 wt.% amphibolite, 30 wt.% glass cullet.). The EDS analysis confirms the presence of a crystalline phase represented by pyroxenes. This is very beneficial from the mechanical properties point of view of the obtained glass-ceramic materials from the CAS system [24,25,26].

As part of the research, Vickers microhardness mechanical tests were realized. The average results from 10 measurement points are presented in Table 4. It was found that the melt obtained from glass 1 (100% basalt) has the highest hardness. This can be explained by the fact that glass containing 100% basalt has the highest content of MgO, which affects the high values of glass hardness and strength [43,44]. For the melts subjected to the crystallization process, it was observed that the microhardness of the glass-ceramic materials grew with the increase in the annealing temperature, which was caused by the presence and amount of crystalline phases formed in the annealed melts. The samples annealed at 1000 °C have the highest microhardness values, which confirms that the formed crystalline phases and their quantity improve the hardness of the investigated materials. The acidity modulus (Mk), a significant factor in assessing the mineral composition of glass and an essential parameter from the mineral fibre production point of view, makes it possible to evaluate the basic properties of the raw material used in glass production. This factor is determined as the weight ratio of all acidic oxides (silica, titanium oxide, and aluminium oxide) to the sum of basic oxides (calcium, sodium, potassium, iron, and magnesium oxide) [45,46]. The following relationship (1) was used to determine the acidity modulus employing the determined chemical composition (Table 2). The obtained M_k_ results are summarized with microhardness tests in Table 4.

(1) $$M_{k}=\frac{\sum {SiO}_{2},{Al}_{2}O_{3},{TiO}_{2}}{\sum CaO,MgO,FeO,{Na}_{2}O,K_{2}O,{Fe}_{2}O_{3}}$$

The correct M_k_ value for mineral wool production (mineral fibres, melting, and defibering) is M_k_ > 1.2 [39]. All of the raw material sets meet this condition, although the variable share of amphibolite and modification with glass cullet increase the M_k_. According to a study by Du P., as the acidity factor increases, the fibre formation temperature, fibre diameter, and the content of non-fibrous parts increase [47]. 

## 4. Conclusions

This study investigates the microstructure (SEM-EDS), phase analysis (FTIR spectroscopy, XRD), and mechanical properties of the SiO_2_-Al_2_O_3_-CaO-MgO-Fe_2_O_3_ multicomponent system glass materials. Based on this research, the proposed raw material sets can successfully find application in producing mineral fibres and novel glass-ceramic materials. Spectroscopic studies of the melted sets revealed the presence of three leading absorption bands in the following ranges: 1200–800 cm^−1^, 800–600 cm^−1^, and 600–400 cm^−1^. After the controlled crystallization process, an increase in the half-width of the band in the 1200–800 cm^−1^ range was observed, confirming the depolymerization of the glass bond and a decrease in the number of Si-O-Si, Si-O-Al bonds. In contrast, the band at about 870 cm^−1^ and 970 cm^−1^ separates in two. The change in the width of the bands and their separation suggest the presence of crystalline phases and a change in the structure of the materials—the formation of crystalline phases during the crystallization process of glasses. These phases are represented by pyroxenes (diopside), whose bands are located at a maximum of 865 cm^−1^, which was confirmed by X-ray and SEM/EDS phase and microstructural results. The melt obtained from glass set 1 (100 wt.% basalt) has the highest hardness owing to the increased CaO, MgO content. For the melts subjected to crystallization, it was observed that the microhardness of glass-crystalline materials rises with increasing annealing temperature. The samples annealed at 1000 °C have the highest mechanical properties (microhardness), which confirms that the crystalline phases formed and their amount improves the hardness of the studied materials. Unfortunately, due to an excessively high acid modulus, the glasses obtained are not quite optimal materials suitable for producing mineral fibres. However, the results obtained will be the preliminary base for further studies in which the authors investigate the production of porous, insulating glass-ceramics materials and mineral fibres.

## Figures and Tables

**Table 1:** Raw material sets used for melting of multicomponent glasses (%).

Sets	Raw Material (%)		
	Basalt	Amphibolite	Glass Cullet
Set 1	100	-	-
Set 2	50	40	10
Set 3	50	30	20
Set 4	50	20	30

**Table 2:** Chemical composition of SiO_2_-Al_2_O_3_-CaO-MgO-Fe_2_O_3_ multicomponent glasses after melting sets 1–4.

Glass	The Chemical Composition of the Glasses								
	SiO_2_	Al_2_O_3_	CaO	MgO	Fe_2_O_3_	Na_2_O	K_2_O	TiO_2_	MnO
1	40.23	13.07	9.39	10.66	10.69	5.04	0.98	2.68	0.16
2	46.80	13.16	9.28	8.12	9.60	6.33	0.85	2.06	0.15
3	48.42	11.80	9.27	7.67	8.29	7.76	0.82	1.89	0.13
4	50.04	10.43	9.26	7.22	7.31	9.20	0.79	1.71	0.11

**Table 3:** The percentage of the crystalline phase calculated according to Rietveld’s least squares approach.

Glass	Degree of Crystallization (%)		
	800 °C/2 h	900 °C/2 h	1000 °C/2 h
1—100% basalt	2.6	7.2	14.8
2—50% basalt, 40% amphibolite, 10% glass cullet	3.5	16.3	21.1
3—50% basalt, 30% amphibolite, 20% glass cullet	4.3	17.2	21.2
4—50% basalt, 20% amphibolite, 30% glass cullet	14.8	21.5	27.3

**Table 4:** Microhardness and acidity modulus M_k_ parameter of SiO_2_-Al_2_O_3_-CaO-MgO-Fe_2_O_3_ multicomponent glasses after melting and crystallization processes.

M_k_ Parameters				
Melted Glass	Glass 1	Glass 2	Glass 3	Glass 4
	1.52	1.83	1.84	1.84
	Microhardness HV0.05			
	753	670	625	631
Annealed Glass	Microhardness HV0.05			
800 °C/2 h	835	751	659	638
900 °C/2 h	913	823	736	767
1000 °C/2 h	946	856	896	859

## Data Availability

The data presented in this study are available on request from the corresponding author. The data are not publicly available due to the possibility of use in further research.

## References

[1] Miao Z., Xingna L., Zhen C., Ji W., Wenhua S. (2014). Experimental study of the heat flux effect on combustion characteristics of commonly exterior thermal insulation materials. Procedia Eng..

[2] Chin D.D., Yahya M.N., Din N.B., Ong P. (2018). Acoustic properties of biodegradable composite micro-perforated panel (BC-MPP) made from kenaf fibre and polyactic acid (PLA). Appl. Acoust..

[3] Zhang D., Yu J., Wu H., Jaworska B., Ellias B., Li V. (2020). Discontinuous micro-fibers as intrinsic reinforcement for ductile Engineered Cementitious Composites (ECC). Compos. Part B.

[4] Ivanitskii S.G., Gorbachev G.F. (2011). Continuous basalt fibers: Production aspects and simulation of forming processes. I. State of the art in continuous basalt fiber technologies. Sov. Powder Met. Met. Ceram..

[5] Perevozchikova V., Pisciotta A., Osovetsky B.M., Menshikov E.A., Kazymov K.P. (2014). Quality evaluation of the Kuluevskaya basalt outcrop for the production of mineral fiber, Southern Urals, Russia. Energy Procedia.

[6] Scheffler C., Förster T., Mäder E., Heinrich G., Hempel S., Mechtcherine V. (2009). Aging of alkali-resistant glass and basalt fibers in alkaline solutions: Evaluation of the failure stress by Weibull distribution function. J. Non-Cryst. Solids.

[7] Grigonis M., Lipinskas D., Maciulaitis R., Jocius V. Fire resistance and reaction to fire tests of buildings construction. Proceedings of the 10th International Conference.

[8] Wu Z., Wang X., Liu J., Chen X. (2020). 13—Mineral fibres: Basalt. Handbook of Natural Fibres.

[9] Elbakian A., Sentyakov B., Božek P., Kuric I., Sentyakov K. (2018). Automated separation of basalt fiber and other earth resources by the means of acoustic vibrations. Acta Montan. Slovaca.

[10] Buratti C., Moretti E., Belloni E., Agosti F. (2015). Thermal and acoustic performance evaluation of new basalt fiber insulation panels for buildings. Energy Procedia.

[11] Farouk M., Soltan A., Schlüter S., Hamzawy E., Farrag A., El-Kammar A., Yahya A., Pollmann H. (2021). Optimization of microstructure of basalt-based fibers intended for improved thermal and acoustic insulations. J. Build. Eng..

[12] Zhao D.W., Zhang Z.T., Tang X.L., Liu L.L., Wang X.D. (2014). Preparation of slag wool byintegrated waste-heat recovery and resource recycling of molten blast furnace slags: From fundamental to industrial application. Energies.

[13] Han B., Du P., Zhang Y., Xing H., Wei G., Wang H. (2020). Influence of polyvinyl alcohol–glutaraldehyde on properties of thermal insulation pipe from blast furnace slag fiber. High Temp. Mater. Process..

[14] Li J., Liu W.X., Zhang Y.Z., Yang A.M., Zhao K. (2014). Research on modifying blast furnace slag as a raw material of slag fiber. Mater. Manuf. Process..

[15] Tang X.-L., Zhang Z.-T., Guo M., Zhang M., Wang X.-D. (2011). Viscosities Behavior of CaO-SiO_2_-MgO-Al_2_O_3_ Slag with Low Mass Ratio of CaO to SiO_2_ and Wide Range of A1_2_O_3_ Content. J. Iron Steel Res. Int..

[16] Holland W., Beall G. (2002). Glass-Ceramic Technology.

[17] Blissett R.S., Rowson R.A. (2012). A Review of the Multicomponent Utilization of Coal Fly Ash. Fuel.

[18] Asquini L., Furlani E., Bruckner S., Maschio S. (2008). Production and characterization of sintered ceramics from paper mill sludge and glass cullet. Chemosphere.

[19] Gao H.T., Liu X.H., Chen J.Q., Qi J.L., Wang Y.B., Ai Z.R. (2018). Preparation of glass-ceramics with low density and high strength using blast furnace slag, glass fiber and water glass. Ceram. Int..

[20] Bai H., Zhang X., Cang D., Zhao L., Wei W. (2015). Synthesis of steel slag ceramics: Chemical composition and crystalline phases of raw materials. Int. J. Miner. Metall. Mater..

[21] Erol M., Küçükbayrak S., Ersoy-Meriçboyu A. (2007). Production of glass-ceramics obtained from industrial wastes by means of controlled nucleation and crystallization. Chem. Eng. J..

[22] Rezvani M., Eftekhari-Yekta B., Solati-Hashjin M., Marghussian V. (2005). Effect of Cr2O3, Fe2O3 and TiO2 nucleants on the crystallization behaviour of SiO_2_-Al_2_O_3_-CaO-MgO(R2O) glass-ceramics. Ceram. Int..

[23] Chinnam R.K., Francis A.A., Will J., Bernardo E., Boccaccini A.R. (2013). Functional glasses and glass-ceramics derived from iron rich waste and combination of industrial residues. J. Non-Cryst. Solids.

[24] Ayala Valderrama D.M., Gómez Cuaspud J.A., Roether J.A., Boccaccini A.R. (2019). Development and Characterization of Glass-Ceramics from Combinations of Slag, Fly Ash, and Glass Cullet without Adding Nucleating Agents. Materials.

[25] Toya T., Kameshima Y., Yasumori A., Okada K. (2004). Preparation and properties of glass-ceramics from wastes (Kira) of silica sand and kaolin clay refining. J. Eur. Ceram. Soc..

[26] Park Y.J., Moon S.O., Heo J. (2003). Crystalline phase control of glass ceramics obtained from sewage sludge fly ash. Ceram. Int..

[27] (2015). Standard Specification for Gypsum Plasters.

[28] Snellings R., Mertens G., Elsen J. (2014). Supplementary Cementitious Materials. Rev. Miner. Geochem..

[29] Yunlong L., Fu W., Hanzhen Z., Qilong L., Laibao L. (2022). Preparation and characterization of glass-ceramics with granite tailings and titanium-bearing blast furnace slags. J. Non-Cryst. Solids Vol..

[30] Luo Y.L., Wang F., Liao Q.L., Liu LBWang Y.L., Zhou J.J., Xu Y.L., Zhu H.Z., Gu Y.X. (2021). Effect of TiO_2_ on crystallization kinetics, microstructure and properties of building glass-ceramics based on granite tailings. J. Non-Cryst. Solids.

[31] Santaella L.E., Correa R.S. (2004). Comportamiento de concreto con bajos porcentajes de ceniza volante (termo paipa IV) y agua constante. Cienc. E Ing. Neogranadina.

[32] Vianchá G., Roldan P.R. (2007). Propuesta Para la Utilización de Cenizas Volantes Como Adición en la Fabricación de Cemento Tipo I en la Planta Cementera de Holcim Colombia S.A.

[33] Rawlings R.D., Wu J.P., Boccaccini A.R. (2006). Glass-ceramics: Their production from wastes—A Review. J. Mater. Sci..

[34] Ojovan W., Juoi J.M., Boccaccini A.R. (2008). Glass Composite Materials for Nuclear and Hazardous Waste Immobilisation. MRS Online Proc. Libr. Arch..

[35] Savvilotidou V., Kritikaki A., Stratakis A., Komnitsas K., Gidarakos E. (2019). Energy efficient production of glass-ceramics using photovoltaic (P/V) glass and lignite fly ash. Waste Manag..

[36] Standard Test Method for Assignment of the Glass Transition Temperature by Thermomechanical Analysis. https://www.astm.org/e1545-22.html.

[37] Sitarz M. (2011). The structure of simple silicate glasses in the light of Middle Infrared spectroscopy studies. J. Non-Crystalline Solids.

[38] Kucharczyk S., Sitarz M., Zajac M., Deja J. (2018). The effect of CaO/SiO_2_ molar ratio of CaO-Al_2_O_3_-SiO_2_ glasses on their structure and reactivity in alkali activated system. Spectrochim. Acta A Mol. Biomol. Spectrosc..

[39] Bayazit M., Isik I., Cereci S., Issi A., Genc E. FT-IR spectroscopic analysis of potsherds excavated from the first settlement layer of kuriki mound, turkey. Proceedings of the International Conference on Ceramics, International Journal of Modern Physics: Conference Series.

[40] Grelowska I., Kosmal M., Reben M., Pichniarczyk P., Sitarz M., Olejniczak Z. (2016). Structural and thermal studies of modified silica-strontium-barium glass from CRT. J. Mol. Struct..

[41] RRUFF. https://rruff.info/Wollastonite/R040131.

[42] Öveçoğlu M., Kuban B., Özer H. (1997). Characterization and crystallization kinetics of a diopside-based glass-ceramic developed from glass industry raw materials. J. Eur. Ceram. Soc..

[43] Jha P., Singh K. (2015). Effect of Field Strength and Electronegativity of CaO and MgO on Structural and Optical Properties of SiO_2_–K_2_O-CaO-MgO Glasses. Silicon.

[44] Kjeldsen J., Smedskjaer M.M., Potuzak M., Yue Y. (2015). Role of elastic deformation in determining the mixed alkaline earth effect of hardness in silicate glasses. J. Appl. Phys..

[45] Krenev V.A., Kondakov D.F., Pechenkina E.N., Fomichev S.V. (2020). Modification of the Composition of Gabbro-Basalt Raw Materials during melting in an oxidizing, Inert, or rediusing atmosphere. Glass Ceram..

[46] Fomichev S.V., Babievskava I.Z., Noskova N.P., Krenev V.A. (2010). Evaluation and modification of the initial composition of gabro-basalt rocks for mineral-fiber fabrication and stone casting. Inorg. Mater..

[47] Du P., Zhang Y., Long Y., Xing L. (2022). Effect of the Acidity Coefficient on the Properties of Molten Modified Blast Furnace Slag and Those of the Produced Slag Fibers. Materials.

